# Reply to Davison, G. Comment on “Huijghebaert et al. Does Trypsin Oral Spray (Viruprotect^®^/ColdZyme^®^) Protect against COVID-19 and Common Colds or Induce Mutation? Caveats in Medical Device Regulations in the European Union. *Int. J. Environ. Res. Public Health* 2021, *18*, 5066”

**DOI:** 10.3390/ijerph20010631

**Published:** 2022-12-30

**Authors:** Suzy Huijghebaert, Guido Vanham, Myriam Van Winckel, Karel Allegaert

**Affiliations:** 1 Independent Researcher, 1310 La Hulpe, Belgium; 2 Department of Virology, Institute of Tropical Medicine, Nationale Straat 155, 2000 Antwerp, Belgium; 3 Department of Paediatrics, Ghent University Hospital and Ghent University, Corneel Heymanslaan 10, 9000 Ghent, Belgium; 4 Department of Development and Regeneration, KU Leuven, Herestraat 49, 3000 Leuven, Belgium; 5 Department of Pharmacy and Pharmaceutical Sciences, KU Leuven, Herestraat 49, 3000 Leuven, Belgium; 6 Department of Hospital Pharmacy, Wytemaweg Hospital Pharmacy, Postbus 2040, Erasmus MC, 3075 CE Rotterdam, The Netherlands

We have read the comment from Davison with great interest [1]. We are grateful that he highlighted and reported these errors on our end, and regret to inform the readership that the paper on trypsin oral spray indeed contains errors [2]. These errors relate to the misinterpretation of one study that reported on the use of ColdZyme mouth spray in endurance athletes and its impact on the duration of upper respiratory tract infection (URTI) symptoms [3]. 

In essence, we initially interpreted this study as a prophylaxis intervention study, as this was suggested as one of the aims of this study (URTI incidence) and mentioned as the first outcome variable at the end of the introduction. Although this specific study still has limitations such as the use of parametric descriptive statistics for non-normally distributed values (standard deviations similar to the mean values), the elimination of all episodes <3 days, or the difference in adherence between both winter seasons, there are also strengths such as the assessment of adherence, the use of the Jackson score and the daily training logs (be it that data were reported in hours/week) [3]. 

In response to the statements of Davison in his letter, we hereby provide the following statements.

1. Related to the statement ‘in a smaller open label study in athletes, the proportions of virus-protected persons per treatment group were not released, but there was no significant difference in number of common cold episodes between the ColdZyme exposed and controls’.
2. Technically, this statement remains correct (as all cases <3 days were not considered), but we fully agree that our wording suggests the concept of a prophylaxis study. We confirm the fact that this is a treatment-driven study, to be used ‘at first self-perceived signs of URTI’. This is clearly stated in the methods section, in the last sentence of the introduction, ‘to assess the efficacy of ColdZyme on URTI incidence, symptom ratings and missed or reduced training in competitive endurance athletes…’ As efficacy on URTI incidence is listed first, this suggests that this was the primary outcome variable. We are sorry for this error and suggest adapting the sentence to, ‘*in a smaller open label study in athletes, there was no significant difference in the number of common cold episodes between the ColdZyme exposed and controls, as the study was focused on URTI treatment as soon as self-perceived symptoms began*’.
3. Related to the statement, ‘the study mixing (unweighted) data obtained from winter seasons over 2 years claimed a reduction of 3.5 days with ColdZyme exposed cases versus controls’.
4. We understand that this statement reads as somewhat negative in the perception of Davison; therefore, we suggest adapting ‘claimed’ to ‘observed’ so that this sentence reads as the following, ‘*the study mixing (unweighted) data obtained from winter seasons over 2 years observed a reduction of 3.5 days with ColdZyme-exposed cases versus controls*’. Unfortunately, this is not a placebo-controlled trial, and we understand that efforts have been made to balance randomization, be it that history on URTI incidence, cold severity score at entry, or household contacts may have been, in our opinion, other potential relevant covariates.
5. **A**. Associated to this statement, ‘However, patients in the control group felt less need for using Over the Counter (OTC) medication than in the Coldzyme cases (38 versus 48%, not significant) undercutting the relevance of the findings’.
6. We reconfirm the error on our end and confirm that we obtained the numbers the wrong-way-round. We suggest that this sentence should read, ‘*Participants felt the need to use OTC medication for 48% of events in controls, and 38% of events in ColdZyme cases (not significant)*’.
7. **B.** Related to the statement, ‘OTC-rescue medication was felt needed in more common cold episodes in Coldzyme cases than in the control group, debunking the spray’s benefits for treatment of common cold in one study, while another open prospective study claims 23% reduced use of rescue medication with Coldzyme further fueling inconsistent findings’ and in Table 2 ‘are not corroborated by the fact that less subjects in the control group felt need for rescue medications than on Coldzyme (38% versus 48%).
8. As both these comments are related to the 3A comment and to the same error, *we suggest removing both sentences.*
9. Related to this error (we obtained the numbers the wrong-way-round), Table 2 should also be adapted. We copied the initial Table 2 from the Huighebaert et al. paper [2] and have adapted the table section on the Davison study [3]. The former and adapted versions of Table 2 are provided, and the adaptations are discussed below.
10. In essence, we have removed the sentence, ‘*no data on the proportions of persons per treatment group*’ (although this is technically correct, as all URTI events <3 days were not considered). We have adapted the prophylaxis to a curative study design (‘*curative intervention study*’). The sentence ‘…*are not corroborated by the fact that less subjects in the control group felt need for rescue medication than on CZ-MD (38% vs. 48%)*’ has been removed and substituted with ‘*Participants felt the need to use OTC medication for 45% of events in controls, and 38% of events in ColdZyme cases (not significant)*’. Finally, with regard to the statement on other parameters, ‘*No significant effects on training-related outcomes*’ has been adapted to ‘*No significant effects on weekly hours of training, but on the number of days missed*’. Finally, ‘*data on common cold episodes were not or not consistently corrected for compliance versus baseline variables and different treatment periods*’ has been removed.

**Table 2:** *Does Viruprotect/ColdZyme (CZ-MD) protect ‘against’ viruses?* Clinical outcomes as derived from studies assessing CZ-MD and when data are available, compared with placebo, controls or non-use.

***Former version***		
Clinical Study	% Users Calculated or Rated as Protected *	Source Document
Observational study, 2 × 3-month periods, prophylaxis in endurance athletes: Dec 2017–Feb 2018 + Dec 2018–April 2019 (compliance enhanced to 6×/day in 2018–2019) n = 62 CZ-MD n = 61 controls—4–10 h of training/week—	CZ-MD = Control group for number of episodes/person (No data on the proportions of persons per treatment group). Other parameters (e.g., claimed reduction of the CC episode by 3.5 days with CZ-MD versus controls) are not corroborated by the fact that less subjects in the control group felt need for rescue medication than on CZ-MD (38% vs. 48%). No significant effects on training-related outcomes. Data on CC episodes were not or not consistently corrected for compliance versus baseline variables and different treatment periods.	Davison et al. [3]
Revised version		
Clinical Study	% Users Calculated or Rated as Protected	Source Document
Observational study, 2 × 3-month periods, curative intervention study in endurance athletes: Dec 2017–Feb 2018 + Dec 2018–April 2019 (compliance enhanced to 6×/day in 2018–2019) n = 62 CZ-MD n = 61 controls—4–10 h of training/week—	CZ-MD = Control group for number of episodes/person. Other parameters: reduction of CC episodes by 3.5 days with CZ-MD (versus controls). Participants felt the need to use OTC medication for 45% in events in controls, and 38% in events in ColdZyme cases (not significant). No significant effects on weekly hours of training, but on the number of days missed.	Davison et al. [3]

* Calculation of protection = number of persons not developing Rhinovirus infection or CC symp-toms, derived from the total number of subjects treated minus those developing symptoms.

11. Statement: ‘Moreover, the reduction in training load (hours/week) during common cold, the return to normal (training load) and the total number of training days were not significantly different between groups’.
12. Our description of the study refers to Figure 2 of the Davison et al. paper [3]. This figure clearly states that the training load (hours/week) is significantly different from the two weeks before and the three weeks after the common cold week, without any other significant difference between both groups. Reassessing the paper, training load has been quantified by ‘hours/week’, not the number of training days, nor their intensity. This is also explicitly mentioned in the Davison paper: (the number of days on which training was reduced, as a consequence of an episode, was not significantly different between the ColdZyme and Control group (*p* = 0.475) [3].
13. As there might be a nuance difference between ‘the total number of training days and the number of training days on which training was reduced (on duration)’ we suggest adapting the sentence to: ‘*Moreover, the reduction in training load (hours/week) during common cold and the return to normal (training load) and the number of training days on which training was reduced were not significantly different between groups, the average number of training days missed was different*’. However, these data are difficult to interpret as these data are non-normally distributed.

## Conclusions

We would like to reiterate that we are grateful that Davison highlighted and reported on these errors on our end, and regret to inform the readership that the paper on trypsin oral stray indeed contained some specific errors related to one study that was indeed a curative interventional study, and not a prophylaxis study. We have explained that this error likely related to the suggestion in the last sentence of the introduction that the incidence of URTI was the first mentioned outcome variable. 

Following this comment, we have verified all other statements and could not find other inaccuracies in our paper. This also means—further strengthened by our errors—that the overarching conclusion that medical device regulations in the European Union need further optimization still holds. To further illustrate this, the protocol and predefined primary and secondary outcome variables for interventional, randomized trials on medicines have to be in the public domain, although this is not yet the case for studies with these types of medical devices. We want to reiterate that the trypsin oral spray hereby served as an example or case study to illustrate and support this overarching message [2]. 
